# Risk factors for infection and outcomes in infants with neonatal encephalopathy: a cohort study

**DOI:** 10.1038/s41390-024-03157-9

**Published:** 2024-04-02

**Authors:** David Odd, Hemmen Sabir, Simon A. Jones, Chris Gale, Ela Chakkarapani

**Affiliations:** 1 Cardiff University, The School of Medicine, Cardiff, UK; 2 Department of Neonatology and Pediatric Intensive Care, Children’s Hospital, University of Bonn, 53127 Bonn, Germany; 3 Neonatal Medicine, School of Public Health, Imperial College London, London, UK; 4 Translational Health Sciences, Bristol Medical School, University of Bristol, Bristol, UK; 5 St Michael’s Hospital Neonatal Intensive Care Unit, University Hospitals Bristol & Weston NHS Foundation Trust, Bristol, UK

## Abstract

**Background:**

To determine the association between early infection risk factors and short-term outcomes in infants with neonatal encephalopathy following perinatal asphyxia (NE).

**Methods:**

A retrospective population-based cohort study utilizing the National Neonatal Research Database that included infants with NE admitted to neonatal units in England and Wales, Jan 2008–Feb 2018. Exposure: one or more of rupture of membranes >18 h, maternal group B streptococcus colonization, chorioamnionitis, maternal pyrexia or antepartum antibiotics. Primary outcome: death or nasogastric feeds/nil by mouth (NG/NBM) at discharge. Secondary outcomes: organ dysfunction; length of stay; intraventricular hemorrhage; antiseizure medications use.

**Results:**

998 (13.7%) out of 7265 NE infants had exposure to early infection risk factors. Primary outcome (20.3% vs. 23.1%, OR 0.87 (95% CI 0.71–1.08), *p* = 0.22), death (12.8% vs. 14.0%, *p* = 0.32) and NG/NBM (17.4% vs. 19.9%. *p* = 0.07) did not differ between the exposed and unexposed group. Time to full sucking feeds (OR 0.81 (0.69–0.95)), duration (OR 0.82 (0.71–0.95)) and the number of antiseizure medications (OR 0.84 (0.72–0.98)) were lower in exposed than unexposed infants after adjusting for confounders. Therapeutic hypothermia did not alter the results.

**Conclusions:**

Infants with NE exposed to risk factors for early-onset infection did not have worse short-term adverse outcomes.

**Impact:**

- Risk factors for early-onset neonatal infection, including rupture of membranes >18 h, maternal group B streptococcus colonization, chorioamnionitis, maternal pyrexia or antepartum antibiotics, were not associated with death or short-term morbidity after cooling for NE.
- Despite exposure to risk factors for early-onset neonatal infection, infants with NE reached oral feeds earlier and needed fewer anti-seizure medications for a shorter duration than infants with NE but without such risk factors.
- This study supports current provision of therapeutic hypothermia for infants with NE and any risk factors for early-onset neonatal infection.

## Introduction

Globally, neonatal encephalopathy following birth asphyxia (NE) ranks as the second major contributor to death and disability adjusted life years,^1^ with the greatest burden occurring in low and middle-income countries. In these regions, outcomes for infants with NE tend to be even worse when they also have early-onset infection.^2^ Furthermore, therapeutic hypothermia (TH) which is the standard care in high-income countries was not effective in reducing death or disability after NE in low and middle income countries,^3^ which may be related to differences in the characteristics of the infants such as chronic exposure to hypoxia.^3^ or exposure to infection alongside hypoxia.^4^ In preclinical models of gram-negative infection sensitized hypoxia-ischemia, TH was not effective in reducing brain injury.^5^ These findings may dissuade clinicians even in high-income countries from offering TH to infants with NE who may have had exposure to early-onset infection risk factors, as seen in a national cohort study which found a significantly higher prevalence of risk factors for early infection in infants with NE who were not offered TH, compared to infants who were offered TH.^6^

Children who underwent TH for NE in high-income countries, despite having improved outcomes at early childhood,^7^ have lower cognitive,^8^ communication,^9^ attention and visuospatial scores.^10^ compared to control children, at early school-age. One potential explanation for this impact on cognitive function could be exposure to infection or inflammation alongside perinatal asphyxia.^11^ However, data supporting worse outcomes for infants with NE exposed to proven infection or early-onset neonatal infection risk factors in high-income countries are limited and sometimes contradictory. For instance, infants with NE who contract group B streptococcal infection have higher mortality than those with NE alone.^12^ Conversely, proven and probable sepsis complicating NE did not amplify the rate of death and developmental impairments compared to NE without sepsis.^13^ Nevertheless, neonatal sepsis was associated with watershed predominant pattern of injury after NE.^14^ Exposure to risk factors for early-onset neonatal infection such as chorioamnionitis in infants with NE reduced the risk of moderate-severe brain injury and adverse cognitive outcomes.^14^ In contrast, exposure to chronic villitis was associated with impaired neurodevelopment.^15^

The conflicting nature of these findings may stem from factors such as the gestational age of the fetus at which hypoxia-ischemia occurred, and the timing, dosage, and nature of inflammation before the hypoxic-ischemic insult.^16–19^ In clinical settings, it is often impossible to precisely determine the timing of exposure to infection or inflammation and the hypoxic-ischemic insult before birth. Newborn infants exposed to antepartum infection or inflammation are identified using risk factors for early-onset neonatal infection, including exposure to prolonged rupture of membranes, vaginal group B streptococcus colonization, maternal pyrexia or clinically suspected chorioamnionitis.^20^

While previous research has explored the association between culture positive neonatal sepsis and outcomes in NE,^12,13^ the relationship between exposure to risk factors for early-onset neonatal infection and outcomes in NE have not been adequately examined. Because the incidence of NE and exposure to risk factors for infection in high-income countries is low, large population-based cohorts are needed to address this question. Moreover, babies with severe NE may not complete three days of TH due to early instigation of palliative care during TH, and some infants with concerns of having sepsis or exposure to risk factors for infection may not be offered TH.^6^ due to concerns that exposure to infection and hypoxia-ischemia may diminish the neuroprotective effect of TH. Excluding babies with severe NE who were not cooled and underwent palliative care will lead to bias as these babies will have the worst outcomes. Therefore, in this study, we used national population-level data to determine whether exposure to risk factors for early-onset neonatal infection, in infants cooled and not-cooled for NE, was associated with a composite outcome of death, or receiving nasogastric feeds or being nil by mouth at discharge.

## Methods

### Data source

The research population was drawn from all infants admitted to neonatal units in England and Wales between 2008 and 2018, with data entered onto the National Neonatal Research Database (NNRD).^21^ The NNRD uses a designated approved dataset (National Neonatal Data Set).^22^ within the NHS Data Dictionary with all data items cleaned and pseudo-anonymized. Infants who died prior to admission to a neonatal unit are not recorded. This study was approved by the Northwest - Greater Manchester East Research Ethics Committee and the Health Research Authority (18/NW/0377).

### Population and exposure

Infants were included if they were born at or above 36^+0^ weeks gestation; and had a clinician recorded diagnosis of moderate/severe NE. The grade of encephalopathy was defined as the worst level recorded (2 for “moderate hypoxic ischemic encephalopathy”, “moderate neonatal encephalopathy”, “grade 2 hypoxic ischemic encephalopathy”; or 3 for “grade 3 hypoxic ischemic encephalopathy”, “severe hypoxic ischemic encephalopathy”, “severe neonatal encephalopathy”). TH was defined as therapeutic hypothermia / active cooling on the daily data for 2 or more calendar days.

Infants were considered to have been exposed to risk factors for early-onset neonatal infection prior to perinatal asphyxia if at birth, there was evidence of prolonged ruptured membranes (>18 h), a diagnosis of maternal group B streptococcus carriage, chorioamnionitis, intrapartum fever, or the mother received intrapartum antibiotics for possible chorioamnionitis. There were no exclusions at the data extraction stage and the dataset included all infants who were diagnosed with NE and those infants who received TH at neonatal units in England and Wales.

### Outcomes

The primary outcome was a composite of death prior to discharge from the neonatal unit, or receiving nasogastric feeds or no enteral feeds on the last day of neonatal care (as a marker of impaired neurological status). The last day of neonatal care indicates when the infant died or the last day of neonatal care in a neonatal unit in England or Wales regardless of neonatal unit to neonatal unit transfer.

Secondary outcomes included:

- Major organ dysfunction, defined as satisfying any of the two following factors: (i) ventilated, (ii) receiving inhaled nitric oxide or pulmonary vasodilator, (iii) receiving inotropic support, (iv) receiving fresh frozen plasma or cryoprecipitate or platelets transfusion, or (v) renal failure or impairment.
- The length of stay in any neonatal unit, as a continuous measure in days.
- Age (in days) to full sucking feeds; defined as the first day the infant received milk only by breast, bottle or cup feeds.
- The worst grade of intraventricular hemorrhage (IVH) (grade 0–4) as per Papile Classification,^23^ recorded from episodic patient data records.
- Treatment for seizures with phenobarbital, phenytoin, levetiracetam, midazolam, paraldehyde, lidocaine, diazepam or lorazepam during neonatal stay.
- The number of days an infant received anti-seizure medications, and the total number of different antiseizure medications used (from the above list).

Other covariates, and potential confounders were also derived from the infants’ electronic patient record and grouped into two categories; demographic factors (maternal age, parity, birth weight (grams), gestational age (completed gestational weeks), sex, and multiple birth) and clinical factors that may be related to HIE included mode of delivery (emergency caesarean, elective caesarean, forceps/ventouse, or vaginal/unassisted), Apgar scores at 1 and 5 min and lowest cord pH (venous or arterial).

### Statistical analysis

Initially, the characteristics of the population were described, split by their exposure status. Comparisons were made by Chi^2^, *t*-test, or Mann–Whitney *U* test as appropriate. In the univariable analysis we investigated the association between the exposure to risk factors for early-onset neonatal infection and the primary, and secondary outcomes. Ordinal variables were summarized using geometric means (rather than centiles) to distinguish small but potentially important differences due to likely skewed distributions. All comparisons of continuous/ordinal variables were made using Mann–Whitney *U* test. Next, we derived regression models (logistic or ordinal as appropriate) to further investigate the association between exposure to risk factors for early-onset neonatal infection and the outcomes. Models were multi-level (random effects) models for year of birth to compensate for changes in diagnosis rates during the period of the study. Initially, unadjusted (other than year of birth) logistic regression models were derived. Associations were then adjusted for potential covariates by adding the confounders described above to the model in groups of common variables. Sex and multiple birth were included as binary terms, and mode of births as a categorical one (i.e., dummy variables were derived). All other variables were included as continuous terms. Finally, we repeated the analysis, adding an interaction term between TH and infection risk exposure, to examine whether TH alters the association between infection risk and the primary, or secondary outcomes, after adjusting for confounders. Ordinal regression was used for numerical measures (length of stay, days to full sucking feeds, days of anti-seizure medications and number of anti-seizure medications).

### Sensitivity analyses

We repeated the analysis using a multiple imputation technique (chained equations) to investigate any impact of missing exposure, outcome and covariates measures using the whole eligible cohort. Imputation was conducted similarly to our previous work.^24^ Analysis was performed on 50 imputed datasets. All variables used in the regression models were included in the imputation process (Supplementary Table 1). We then repeated the main analysis limited to those children who received therapeutic hypothermia for 2 or more days to examine the impact of exposure to risk factors for neonatal infection in those babies who received a complete course of TH, for the primary and secondary outcomes adjusting for the unit of birth by using both year of birth, and unit of birth as the grouping variable in the random effects model, and adding in the grade of NE (a likely mediator) into the model, and for each of the components of the organ dysfunction variable. Finally, we repeated the main analysis using the number of risk factors for early infection as an ordinal term to derive an OR for each additional factor. All analyses were performed using Stata v14 (Stata Corp).

## Results

**Fig. 1:**
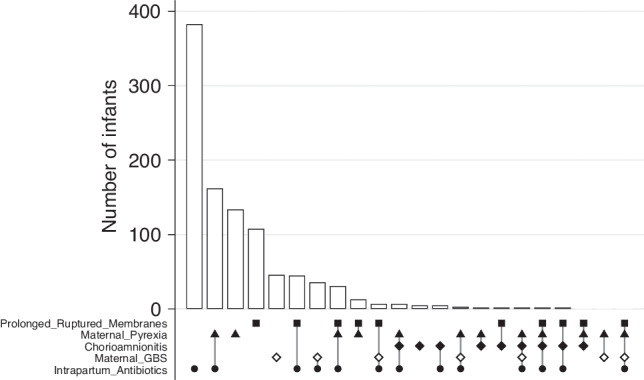
Upset plot showing the number of infants with different combinations of risk factors for early-onset neonatal infection. The upset plot shows the 22 combinations of inflammatory risk factors seen, and order of their relative frequencies. Isolated intrapartum antibiotics was the most common.

The initial cohort contained data on 564,898 infants. Infants without evidence of encephalopathy were removed (*n* = 557,633), leaving 7265 eligible infants of which 998 infants (13.7%) had recorded exposure to risk factors for early-onset neonatal infection. Proportion of infants with different components of risk factors include prolonged rupture of membranes (212 (2.9%)), group B streptococcus colonization (96 (1.3%)), clinically diagnosed chorioamnionitis (28 (0.4%)), maternal pyrexia (359 (4.9%)) and intrapartum antibiotics (686 (9.4%)). Figure 1 shows the 22 combinations of inflammatory risk factors seen, and order of their relative frequencies. Isolated intrapartum antibiotics was the most common, followed by a combination of intrapartum antibiotics and maternal pyrexia. No infants had all 5 risk factors. Exposed infants as compared to those not exposed tended to have higher birthweight, were slightly more mature, were less likely to be born from multiple pregnancies and more likely to be born to primiparous women (Table 1). They were of similar sex and maternal age. Infants exposed to risk factors for early-onset neonatal infection had a different profile of mode of delivery (being more likely to be born by vaginal forceps delivery and by elective LSCS, *p* < 0.001), had higher cord pH measures (*p* < 0.001) and a lower proportion of grade 3 NE (*p* < 0.003), but similar Apgar scores, compared to unexposed infants (Table 1).

**Table 1:** Demographics of the study population, split by their exposure status (*n* = 7265).

Measure	*n*	Non-exposed (*n* = 6267)	Exposed to risk factors for infection (*n* = 998)	*p*-value
Birth Weight	7155	3349 (611)	3455 (594)	<0.001
Gestational Age	7265	39.4 (1.6)	39.5 (1.6)	0.0236
Male	7156	3380 (54.8%)	568 (57.2%)	0.166
Multiple Birth	7152	171 (2.8%)	14 (1.4%)	0.012
Maternal Age	7224	30.3 (6.0)	30.2 (6.0)	0.771
Parity	6230	1 (0–2)	0 (0–1)	<0.001
Mode of Delivery	7016			<0.001
Emergency LSCS		2066 (34.3%)	219 (22.3%)	
Elective LSCS		2877 (47.7%)	541 (55.0%)	
Forceps/Ventouse		76 (1.3%)	11 (1.1%)	
Vaginal (Unassisted)		1013 (16.8%)	213 (21.7%)	
Apgar (1 min)	6769	2 (0–3)	2 (1–3)	0.3585
Apgar (5 min)	6788	4 (2–6)	4 (2–6)	0.7364
Lowest cord pH	5724	6.98 (6.82–7.13)	7.02 (6.88–7.18)	<0.001
Grade 3 NE	7265	2458 (39.2%)	342 (34.3%)	<0.003

Values are mean (SD), median (IQR) or *n* (%).

Statistical comparisons made by *t*-test, Mann–Whitney *U* or Chi^2^ test as appropriate.

In the univariable analysis, there was weak evidence that infants exposed to risk factors for early-onset neonatal infection were less likely to die or have nasogastric feeds or no feeds on their last day of neonatal care (20.3% vs 23.1%, *p* = 0.052) (Table 2). For the secondary outcomes, infants exposed to risk factors for early-onset neonatal infection compared with unexposed infants appeared to reach suck feeds faster (8.4 vs 9.5 days, *p* = 0.0006), had less days of antiseizure medications (2.9 vs 3.8, *p* = 0.0056), and fewer different antiseizure medications (0.8 vs 0.9, *p* = 0.0079) (Table 2). There was less evidence for any difference in major organ dysfunction, overall length of stay, grade of IVH and treatment for seizures; although for all measures the infants exposed to risk factors for early-onset neonatal infection appeared to have lower/better measures than unexposed infants (Table 2).

**Table 2:** Univariable associations between exposure to risk factors for early onset neonatal infection and outcomes.

Measure	*N*	Non-exposed (*n* = 6267)	Exposed group (*n* = 998)	*P*
Primary Outcome				
Dead or NG tube fed/NBM on last day of neonatal care	7265	1449 (23.1%)	203 (20.3%)	0.052
Dead	7265	877 (14.0%)	128 (12.8%)	0.321
NG or NBM on last day of neonatal care	7264	1246 (19.9%)	174 (17.4%)	0.070
Secondary Outcomes				
Major Organ Dysfunction	7057	3088 (50.9%)	476 (47.9%)	0.081
Length of stay (days)	7264	14.3 (0.2)	12.2 (0.4)	0.0824
Time to full sucking feeds (days)	5015	9.5 (0.1)	8.4 (0.2)	0.0006
Grade of IVH (0–4)	2328	18 (0.9%)	0 (0.0%)	0.483
Seizure treatment received	7264	3616 (57.7%)	547 (54.8%)	0.086
Total days of antiseizure medications	7264	3.8 (0.1)	2.9 (0.2)	0.0056
Number of antiseizure medications	7264	0.9 (0.0)	0.8 (0.0)	0.0079

Summary values are geometric means (SE) or *n* (%) as appropriate. Statistical comparisons made by Mann–Whitney *U* or Chi^2^ test as appropriate.

*NG* nasogastric, *NBM* nil by mouth, *IVH* intraventricular hemorrhage.

In the unadjusted multi-level (random effects) model, infants exposed to risk factors for early-onset neonatal infection compared to unexposed infants had weak evidence for a reduction in the primary outcome (OR 0.86 (0.73–1.02), *p* = 0.078), and this remained similar in the model adjusting for demographic factors (OR 0.84 (0.70–1.01), *p* = 0.064) (Table 3). In the final model, adjusted for demographic and clinical factors, there was little evidence to support an association, although the result was less precise due to smaller numbers and the absolute point effect was similar (OR 0.87 (0.71–1.08), *p* = 0.215) (Table 3).

**Table 3:** Multivariable associations between exposure to risk factors for early-onset neonatal infection and outcomes.

Measure	Unadjusted			Adjusted for demographic^a^ factors^b^			Adjusted for demographic^a^ and clinical factors^b^			Adjusted for demographic^a^ and clinical factors^b^ and unit of birth^c^		
	*n*	OR (95% CI)	*p*	*n*	OR (95% CI)	*p*	*n*	OR (95% CI)	*p*	*n*	OR (95% CI)	*p*
Primary Outcome												
Dead or NG/NBM on last day of neonatal care	7265	0.86 (0.73–1.01)	0.078	6113	0.84 (0.70–1.01)	0.064	4687	0.87 (0.71–1.08)	0.215	4687	0.86 (0.70–1.07)	0.171
Dead	7265	0.91 (0.74–1.11)	0.332	6113	0.92 (0.75–1.14)	0.467	4687	1.01 (0.78–1.30)	0.953	4687	0.99 (0.77–1.28)	0.953
NG on last day of neonatal care	7264	0.86 (0.72–1.02)	0.082	6113	0.84 (0.70–1.02)	0.076	4687	0.86 (0.69–1.08)	0.188	4687	0.86 (0.69–1.07)	0.178
Secondary Outcomes												
Major Organ Dysfunction	7057	0.89 (0.78–1.01)	0.081	5966	0.92 (0.80–1.06)	0.249	4573	0.90 (0.76–1.06)	0.193	4573	0.89 (0.75–1.06)	0.192
Length of stay	7264	0.90 (0.81–1.01)	0.083	6112	0.93 (0.82–1.05)	0.223	4686	0.89 (0.77–1.01)	0.079	4686	0.90 (0.78–1.03)	0.121
Time to full sucking feeds	5015	0.79 (0.69–0.90)	<0.001	4250	0.83 (0.72–0.95)	0.009	3316	0.80 (0.68–0.94)	0.007	3316	0.81 (0.69–0.95)	0.009
Severe IVH (grade3/4)	2328	0.95 (0.48–1.86)	0.870	2062	0.72 (0.32–1.60)	0.418	1595	0.77 (0.34–1.76)	0.543	1595	0.74 (0.33–1.70)	0.484
Seizure treatment received	7264	0.91 (0.77–1.04)	0.178	6113	0.90 (0.78–1.04)	0.148	4687	0.86 (0.74–1.02)	0.078	4687	0.86 (0.73–1.02)	0.083
Days of antiseizure medications	7264	0.86 (0.76–0.97)	0.017	6113	0.84 (0.74–0.96)	0.011	4687	0.82 (0.71–0.95)	0.007	4687	0.82 (0.71–0.95)	0.009
Number of anti-seizure medications	7264	0.86 (0.76–0.97)	0.015	6113	0.86 (0.75–0.98)	0.023	4687	0.83 (0.71–0.96)	0.014	4687	0.84 (0.72–0.98)	0.025

Multi-level modeling by year of birth. Ordinal regression was used for numerical measures (Length of stay, days to full sucking feeds, days of anti-seizure medications and number of anti-seizure medications).

^a^Adjusted for maternal age, parity, birthweight, gestation, sex, multiple birth.

^b^Adjusted for mode of birth, Apgar scores at 1 and 5 min and lowest cord pH.

^c^Adjusted for unit of birth.

For the secondary outcomes, results were also consistent with the univariable results, and all statistical associations weakened in the final model when adjusted for clinical (and potentially causal) factors; although point estimates remained similar. There did however remain evidence that time to full sucking feeds (OR 0.80 (0.68–0.94)), duration of antiseizure medications (OR 0.82 (0.71–0.95)) and the number of antiseizure medications (OR 0.83 (0.71–0.96)) were lower in infants exposed to risk factors for early-onset neonatal infection, even after adjusting for demographic and clinical factors (Table 3). There was little evidence that the relationship between exposure and any of the outcomes (primary or secondary) was modified by the grade of NE (Fully adjusted model, all *p*_interaction_ > 0.05).

### Sensitivity analyses

Repeating the model with a multiple imputation model (*n* = 7265, unadjusted OR (0.86 0.73–1.02); fully adjusted OR 0.85 (0.71–1.02) and restricted to those infants who received therapeutic hypothermia for 2 or more days (*n* = 5206, unadjusted OR (0.87 (0.71–1.05)); fully adjusted OR 0.86 (0.67–1.09)) gave compatible results to the main analysis. Repeating the analysis, deriving an OR for the number of increasing risk factors for early infection, gave compatible results to the main analysis (unadjusted OR 0.90 (0.81–1.01); fully adjusted OR 0.95 (0.83–1.10)), as did additional adjusting for unit of birth (unadjusted OR 0.85 (0.72–1.01); fully adjusted OR 0.86 (0.70–1.07)). Adjusting for the unit of birth in the secondary analysis gave similar results (Table 3), for the time to full sucking feeds (OR 0.81 (0.69–0.95)); days OR 0.82 (0.71–0.95) and number of anti-seizure medications OR 0.84 (0.72–0.98). An analysis adding the grade of NE to the final regression model, also showed no evidence of worse primary outcomes in the infants exposed to risk factors for early neonatal infection (OR 0.99 (0.78–1.25)). There was little evidence of an association between exposure to risk factors for early neonatal infection and (in isolation) ventilation, nitric oxide, pulmonary vasodilator, inotropes or clotting products/platelets in the infants, in either the univariable (Supplementary Table 2) or multivariable (Supplementary Table 3) analyses. There was some evidence that infants exposed to risk factors for early neonatal infection had lower levels of renal impairment (5.5% vs 7.3%) in the unadjusted results, but this weakened slightly in the final model (OR 0.71 (0.50–1.01)).

## Discussion

Over a nearly 10-year period, more than 7000 infants were admitted to neonatal units with moderate or severe hypoxic-ischemic encephalopathy across England and Wales. The 13.7% of these infants who were exposed to risk factors for early-onset neonatal infection had less severe encephalopathy, less evidence of acidosis, and did not appear to have worse outcomes compared to infants presenting in a similar way but without evidence of exposure to risk factors for early-onset neonatal infection, even after adjusting for clinical severity including level of acidosis. They were less likely to die or have evidence of impaired oromotor skills at discharge, and reached sucking feeds faster and received less antiseizure medications. These results were similar after adjusting for the measures of asphyxia but results in this analysis were imprecise and interpretation is limited. However, even after these adjustments, exposure to risk factors for early-onset neonatal infection did not appear associated with worse outcomes compared with unexposed infants in some domains, such as time to full oral feeds. Indeed, even after including the clinical consequences of the perinatal asphyxia (the grade of HIE, a possible mediatory of outcome) into the model, there was little evidence for an association between exposure to risk factors of neonatal infection and adverse outcome.

We used exposure to risk factors for early-onset neonatal infection to quantify exposure to infection or inflammation before perinatal asphyxia occurred. These risk factors captured maternal infection, risk for fetal infection and clinically defined chorioamnionitis. Our definition of exposure aligned with the NICE guideline for early-onset neonatal sepsis.^20^ and with the recommendations of Strengthening the Reporting of Observational Studies in Epidemiology for Newborn Infection.^25^ Typically, when clinicians are considering commencing TH in infants with NE before 6 h of life, they will only have information on whether the infants were exposed to risk factors for early-onset neonatal infection and not results of blood inflammatory markers or blood cultures. In the large randomized controlled cooling trials the rate of perinatal infection was not different between the cooled and non-cooled groups.^7^ However, perinatal infection is defined as evidence of a positive blood culture.^26^ In most neonatal cases, blood cultures remain negative despite strong evidence for perinatal infection. This is mainly due to limitations in blood volumes being used for blood culture incubation.^26^ There is strong pre-clinical evidence showing that the inflammation caused by exposure to bacteria or viruses before birth significantly induces a pro-inflammatory cytokine release, and lowers the threshold for hypoxia-ischemia associated brain injuries, despite the detectability of bacteria in blood cultures.^27^ Our findings are counter to findings from the TH in acute inflammation followed by hypoxia-ischemia animal models; in pre-clinical small and large animal models, where the onset of inflammation induced by gram negative endotoxin LPS prior to hypoxia-ischemia (so called “pre-sensitization”) is exactly defined, cooling failed to be neuroprotective.^5^ These findings contrast with those triggered by a gram positive analogous substance (PAM_3_CSK4), which recorded a beneficial outcome.^28^ LPS and PAM_3_CSK4 activate inflammation through different innate sensing mechanisms (Toll-like receptor [TLR]-4 vs. TLR2/1), suggesting that any neuroprotective effects may be dependent on the nature of the bacterial infection.^28^ In preterm equivalent fetal sheep models, chronic low dose exposure to lipopolysaccharide followed by hypoxic-ischemia reduced brain injury compared to hypoxic-ischemia or lipopolysaccharide exposure alone.^19^ In term gestation equivalent rat models (P9 and P14), hypoxic-ischemic insult after 48 h of lipopolysaccharide exposure reduced the infarct volume compared with hypoxic-ischemic insult group without lipopolysaccharide exposure. The reduction in brain injury was associated with changes in the TLR 4 expression in the rodent brain.^17^ However, none of these animals underwent hypothermia. It is likely that a combination of the above-mentioned experimental paradigms might occur in clinical settings.

In a single center cohort study, NE infants who did and did not undergo TH and were exposed to maternal chorioamnionitis had lower risk of brain injury and adverse outcomes compared to infants without exposure to maternal chorioamnionitis.^14^ Two European centers reported no difference in death, cerebral palsy or developmental delay between infants cooled for NE with and without early neonatal sepsis.^13^ Although these studies align with our findings, in infants cooled for NE, more rigorously defined and longer duration of exposure to chronic villitis was associated with basal ganglia thalami pattern of neonatal brain injury on MRI and adverse neurodevelopmental outcomes at 18–24 months of age.^15^ Interpretation of our findings in the current study have to be considered with caution, as we have a non-specific exposure which will include some infants who are in reality unexposed to infection despite risk factors, and no information regarding time of exposure to infection-inflammation and onset of hypoxia-ischemia. Given that most infants were exposed to intrapartum antibiotics for suspected maternal infection and maternal pyrexia, whether antibiotics may have ameliorated the impact of infection on infants is unknown. Also, hypoxia-ischemia might have been different in individual newborns with regards to acute sentinel events vs. chronic longer lasting phases of hypoxia-ischemia. Further, these conflicting findings may be explained by the differences in the definition of infection and inflammatory exposure, timing of outcome ascertainment (at discharge vs long term), definition of developmental outcomes, smaller sample sizes and small number of infants with the defined exposure.

### Strengths and limitations

We selected nasogastric feeding or no milk feeds at discharge as a marker of short term adverse neurological outcome in this study because oromotor dysfunction in infants with NE is associated with brain injuries on neonatal MRI, in particular brain stem injury,^29^ and also associated with brain dysfunction measured using background activity on electroencephalogram.^30^ This is the first large population representative dataset with nationally reported outcome data without recall bias. Although we lack the long-term outcomes for the infants included in the study, the short-term outcome measure in this study, need for gavage feeds at discharge increased the risk of death or disability when assessed at 18 months of age in a clinical trial of TH.^31^

While we were able to draw our data from a statutory dataset which has been demonstrated to have high completeness for key variables.^32^ and data accuracy that has been validated against prospectively recorded clinical trial data, in any routinely recorded dataset errors in coding are possible and some infants with NE may have been missed.^33^ In addition, we know a proportion of infants with severe neonatal disease are not captured with the NNRD due to early deaths in the delivery room.^34^ and so this work is by necessity performed on a sub-set of infants with NE admitted to neonatal units in the UK. However, it seems likely that errors in coding are likely to be non-differential to the main outcome measured here and so are most likely to reduce the chance of identifying a real association, rather than introduce a false signal. Equally, while we attempted to adjust for the clinical characteristics in the infants, to identify the possible causal impact of the inflammatory process, similar clinical presentations may be seen as a consequence of both pathologies and so interpretation of causality is complicated. We defined major organ dysfunction to include two factors as many infants are likely to be ventilated as part of standard care. Given the lack of complete data in the NNRD on neonatal brain MRI findings or long-term neurodevelopment, our study focused on brain injuries including IVH, which can occur in infants with NE.^35^ and short-term adverse outcomes. We used logistic regression, instead of propensity score matching, as the preferred method of adjusting for confounders, due to the number of confounders and the relative size of the study.^36,37^ Finally, precision is limited in the point estimates, with moderate or severe NE fortunately rare in the UK; and even in the multiple imputation model, a clinically important reduction, or increase, in the primary outcome cannot be excluded (95% confidence intervals of 0.71–1.02).

## Conclusion

In this work, we found little evidence to suggest that infants with moderate or severe NE had a worse short-term outcome of death or adverse oromotor function at neonatal discharge, when they had additional exposure to risk factors for early-onset neonatal infection. Given that we lack robust biomarkers that measure or identify the antepartum infection-inflammation exposure in newborns with NE at the time of delivery, clinicians, faced with an infant with NE requiring TH, should not view exposure to risk factors for early-onset neonatal infection as additional poor prognostic predictors.

## Supplementary information

Supplementary materials

## Data Availability

Data are available upon reasonable request. Applications to use the data used within this project should be made to the Neonatal Data Analysis Unit, Imperial College London.
