# Supplementary material for: Risk factors for infection and outcomes in infants with neonatal encephalopathy: a cohort study
Source: Pediatr Res. 2024 Apr 2;96(3):785–91. doi: 10.1038/s41390-024-03157-9 (PMC11499269; doi:10.1038/s41390-024-03157-9)
Supplement: Supplementary file 1 — Supplementary materials [file 41390_2024_3157_MOESM1_ESM.pdf]

Supplemental Table 1: **Variables in the Multiple Imputation model, their imputation method, and percent of missingness**

**Variable**	**Imputation command**	**% missing**
HIE grade	ologit	0 (0.0%)
Multiple birth	logit	113 (1.6%)
Death or NG	logit	0 (0.0%)
Birth Year	mlogit	0 (0.0%)
Inflammation	logit	0 (0.0%)
Maternal Age	regress	41 (0.6%)
Parity	ologit	1035 (14.3%)
Birthweight	regress	110 (1.5%)
Gestational age	ologit	0 (0.0%)
Sex	logit	109 (1.5%)
Mode of Delivery	mlogit	249 (3.4%)
Apgar Score@1 minute	ologit	496 (6.8%)
Apgar Score@5 minutes	ologit	477 (6.6%)
Apgar Score@10 minute	ologit	1541 (21.2%)

Supplemental Table 2: **Associations between exposure to individual risk factors for early onset neonatal infection and organ dysfunction.**

**Measure**	**N**	**Non-exposed (n=6267)**	**Exposed group (n=998)**	**P**
Ventilation	7264	5284 (84.3%)	846 (84.8%)	0.721
Nitric Oxide	7169	721 (11.7%)	108 (10.9%)	0.473
Pulmonary Vasodilator	6964	602 (10.1%)	90 (9.1%)	0.320
Inotropes	7057	2747 (45.3%)	420 (42.3%)	0.078
Clotting products/platelets	7264	1559 (24.9%)	249 (25.0%)	0.962
Renal Impairment	7265	457 (7.3%)	55 (5.5%)	0.041

Summary values are n (%) as appropriate.
Statistical comparisons made by Chi^2 test as appropriate.

Supplemental Table 3: **Multivariable associations between exposure to risk factors for early-onset neonatal infection and organ dysfunction**

Measure	Unadjusted			Adjusted for demographic* factors**			Adjusted for demographic* and clinical factors**			Adjusted for demographic* and clinical factors** and unit of birth***		
	**n**	**OR (95% CI)**	**p**	**n**	**OR (95% CI)**	**p**	**n**	**OR (95% CI)**	**p**	**n**	**OR (95% CI)**	**p**
Ventilation	7264	1.00 (0.83-1.21)	0.976	6113	1.03 (0.84-1.26)	0.772	4687	0.98 (0.77-1.25)	0.863	4684	0.99 (0.77-1.27)	0.955
Nitric Oxide	7169	0.90 (0.73-1.12)	0.358	6070	0.93 (0.75-1.17)	0.548	4655	0.93 (0.72-1.19)	0.541	4652	0.91 (0.71-1.17)	0.476
Pulmonary Vasodilatory	6964	0.87 (0.69-1.10)	0.234	5896	0.93 (0.73-1.18)	0.529	4518	0.91 (0.70-1.19)	0.502	4516	0.90 (0.69-1.18)	0.453
Inotropes	7057	0.89 (0.77-1.01)	0.078	5966	0.94 (0.81-1.09)	0.416	4573	0.90 (0.76-1.06)	0.225	4570	0.89 (0.75-1.06)	0.202
Clotting products/platelets	7264	0.95 (0.81-1.11)	0.517	6113	0.98 (0.83-1.16)	0.828	4687	0.97 (0.81-1.17)	0.762	4684	1.00 (0.83-1.21)	0.990
Renal Impairment	7265	0.74 (0.56-0.99)	0.045	6113	0.70 (0.51-0.96)	0.026	4638	0.71 (0.50-1.00)	0.048	4635	0.71 (0.50-1.01)	0.053

* Adjusted for maternal age, parity, birthweight, gestation, sex, multiple birth

** Adjusted for mode of birth, Apgar scores at 1 and 5 minutes and lowest cord pH

*** Adjusted for unit of birth

Multi-level modeling by year of birth. Ordinal regression was used for numerical measures (Length of stay, days to full sucking feeds, days of anti-seizure medicine and number of anti-seizure medications).
